# Fulminant Eosinophilic Myocarditis in a Young Adult: Successful Recovery After Extracorporeal Membrane Oxygenation (ECMO) Support and Multisystem Complications

**DOI:** 10.7759/cureus.99032

**Published:** 2025-12-12

**Authors:** Vasileios Leivaditis, Sofien Ayed, Petra Schallmaier, Volker Windmüller, Athanasios Papatriantafyllou, Inna Kammerer, Burghard Schumacher, Manfred Dahm

**Affiliations:** 1 Department of Cardiothoracic and Vascular Surgery, Westpfalz-Klinikum, Kaiserslautern, DEU; 2 Department of Cardiology, Westpfalz-Klinikum, Kaiserslautern, DEU

**Keywords:** cardiogenic shock, endomyocardial biopsy, eosinophilic myocarditis, extracorporeal membrane oxygenation (ecmo), immunosuppressive therapy, ischemic colitis, left ventricular thrombus, multidisciplinary management

## Abstract

Eosinophilic myocarditis (EM) is a rare inflammatory cardiomyopathy with a highly variable clinical presentation, ranging from mild symptoms to fulminant cardiogenic shock. Its diagnosis is challenging due to non-specific clinical findings and requires a high index of suspicion, with endomyocardial biopsy (EMB) serving as the gold standard. Early initiation of immunosuppressive therapy and, in severe cases, mechanical circulatory support (MCS) are essential to improve outcomes. We report the case of a 21-year-old male who presented with progressive dyspnea, chest pain, and hypoxemia. Echocardiography revealed severe left ventricular dysfunction. Coronary angiography excluded obstructive disease, while EMB confirmed EM. Due to cardiogenic shock, veno-arterial extracorporeal membrane oxygenation (ECMO) was implanted on the day of admission. High-dose corticosteroids and azathioprine were initiated, leading to gradual improvement of ventricular function and subsequent ECMO explantation. The course was complicated by left ventricular thrombus formation, which resolved under systemic anticoagulation, and by ischemic colitis requiring right hemicolectomy with ileostomy. Following tracheostomy and prolonged weaning, the patient achieved functional recovery and was discharged on immunosuppressive and anticoagulant therapy. Six months later, surgical restoration of bowel continuity was performed without complications. Follow-up echocardiography demonstrated recovery of left ventricular ejection fraction (LVEF) to 50%, with no evidence of thrombus recurrence. This case highlights the diagnostic and therapeutic challenges of EM, including its fulminant presentation, life-threatening complications, and the critical role of multidisciplinary management. Prompt recognition, biopsy-guided diagnosis, early immunosuppression, MCS when indicated, and careful monitoring for systemic complications can lead to favorable outcomes, even in complex and prolonged clinical courses.

## Introduction

Eosinophilic myocarditis (EM) is a rare and potentially life-threatening inflammatory cardiomyopathy characterized by eosinophilic infiltration of myocardial tissue, often with associated myocyte necrosis, endomyocardial damage, and, in some cases, intracavitary thrombus formation [1]. Clinical presentation of EM is heterogeneous, ranging from mild symptoms such as chest pain or dyspnea to fulminant cardiogenic shock or sudden cardiac death [2].

Diagnosis is challenging and often delayed, because initial symptoms may be non-specific. While imaging modalities, especially cardiac magnetic resonance imaging (CMR), can suggest myocarditis, endomyocardial biopsy (EMB) remains the gold standard for definitive diagnosis [3]. According to recent systematic reviews, EMB is used in the majority of confirmed cases, although a substantial proportion are diagnosed via clinical criteria plus imaging when biopsy is infeasible or high risk [2].

Management generally involves high-dose corticosteroids, sometimes combined with other immunosuppressive agents, and supportive care. Early initiation of therapy is associated with better outcomes, including improved left ventricular ejection fraction (LVEF) and reduction of complications such as thrombus formation [3,4]. In patients presenting with severe hemodynamic compromise, mechanical circulatory support (MCS), including extracorporeal membrane oxygenation (ECMO), has been reported in case series and observational studies as a bridge to recovery [5].

Despite these interventions, the prognosis remains guarded in fulminant cases. Mortality and morbidity remain substantial when diagnosis or treatment is delayed. Functional recovery depends heavily on prompt recognition, aggressive management, and multidisciplinary care [2,4].

The objective of this case report is to describe a fulminant presentation of EM requiring ECMO support, to outline the associated multisystem complications, and to highlight the importance of early biopsy-guided diagnosis, timely immunosuppressive therapy, and multidisciplinary management.

## Case presentation

A 21-year-old male patient with no relevant past medical history was admitted to the emergency department with a three-day history of progressive dyspnea, cough, and chest pain. On presentation, he was severely tachypneic, tachycardic, hypoxemic (oxygen saturation 75% on room air), and agitated.

**Figure 1:**
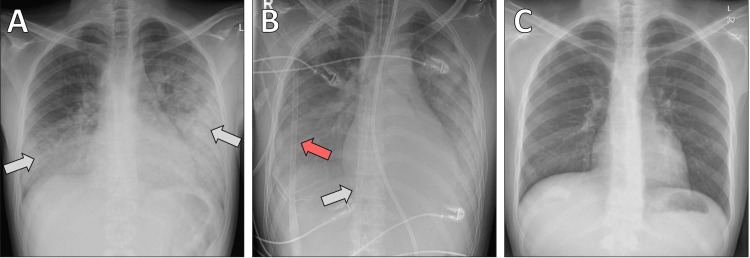
Chest X-ray findings A: on admission, chest radiograph demonstrating advanced bilateral pulmonary edema (white arrows); B: after central implantation of the extracorporeal life support (ECLS) system, arterial cannula in the right axillary artery (red arrow) and venous cannula inserted via the right common femoral vein, extending into the superior vena cava (white arrow); C: on discharge, chest radiograph showing complete resolution of pulmonary pathology with normal findings.

**Figure 2:**
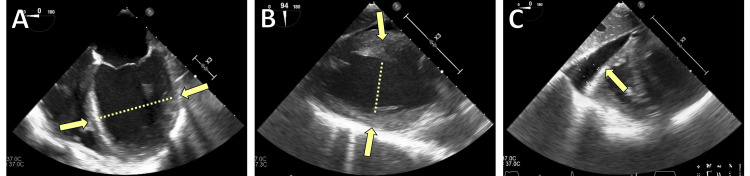
Transesophageal echocardiography A: four-chamber view demonstrating severe left ventricular dilatation (yellow arrows indicating the enlarged ventricular cavity); B: two-chamber view demonstrating severely reduced left ventricular systolic function (yellow arrows marking the hypokinetic regions); C: concomitant pericardial effusion (yellow arrow pointing to the pericardial fluid).

Initial work-up revealed diffuse ST-segment elevations and markedly elevated troponin levels. A chest X-ray revealed bilateral pulmonary edema (Figure 1a). Initial laboratory testing revealed significant leukocytosis, markedly elevated inflammatory markers, and severe myocardial injury (Table 1). Procalcitonin was elevated at 1.9 ng/mL, C-reactive protein (CRP) was 193 mg/L, and high-sensitivity troponin T was 2425 pg/mL. Coronary angiography showed normal coronary arteries, thereby excluding coronary artery disease. Transthoracic echocardiography demonstrated severely reduced LVEF (5-10%) with global hypokinesia (Figure 2).

**Table 1:** Key laboratory findings on admission CRP: C-reactive protein; GFR (CKD-EPI): glomerular filtration rate (chronic kidney disease epidemiology collaboration); LDH: lactate dehydrogenase; CK-MB: creatine kinase-MB; INR: international normalized ratio; aPTT: activated partial thromboplastin time

Parameter	Value	Unit	Normal Range
Leukocytes	19.88	×10³/µL	3.8–10.3
Neutrophils %	88.3	%	40.1–67.0
Lymphocytes %	6.6	%	23.6–48.0
CRP	193	mg/L	<5
Procalcitonin	1.9	ng/mL	<0.05
Sodium	138	mmol/L	136–145
Potassium	3.6	mmol/L	3.4–4.5
Calcium total	2.01	mmol/L	2.15–2.5
Creatinine	1.29	mg/dL	0.7–1.2
Urea	68.7	mg/dL	16.6–48.5
GFR (CKD-EPI)	78.7	ml/min/1.73m²	≥90
Bilirubin total	1.2	mg/dL	0.1–1.2
Glucose	220	mg/dL	60–99
Total protein	5.0	g/dL	6.6–8.7
LDH	1053	U/L	<250
CK total	1081	U/L	20–200
CK-MB	172	U/L	<25
Troponin T	2425	pg/mL	0–14
INR	1.38	-	0.8–1.2
aPTT	28	sec	24–31
D-dimers	11.70	µg FEU/mL	<0.5
Thrombocytes	180	×10³/µL	146–328

**Figure 3:**
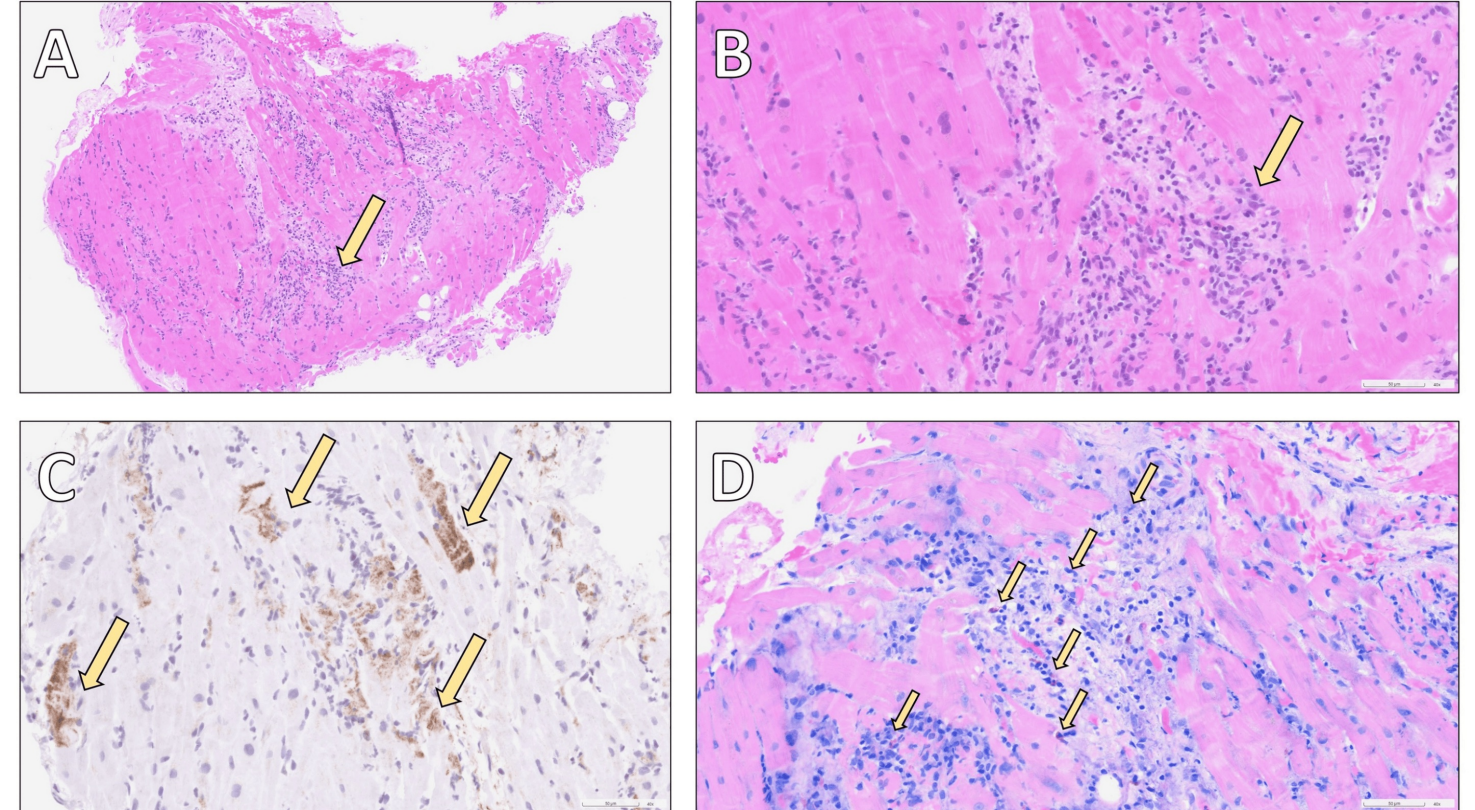
Histopathology of the myocardium Histological section showing dense eosinophilic infiltration associated with inflammation, myocyte necrosis, and interstitial edema. Eosinophilic myocarditis is characterized by predominant perivascular and interstitial eosinophilic infiltrates, with minimal myocyte injury in this specimen. A: hematoxylin–eosin (H&E) staining at ×10 magnification, revealing inflammatory infiltrates within the myocardium (arrow); B: the same staining at ×40 magnification, providing a closer view of the inflammatory process (arrow); C: C4d immunohistochemistry at ×40 magnification, highlighting areas of recent myocardial necrosis (arrows); D: Giemsa staining at ×40 magnification, demonstrating numerous eosinophilic granulocytes within the inflammatory infiltrates (arrows).

Given impending cardiogenic shock, the patient was intubated and placed on central veno-arterial extracorporeal life support (ECLS/ECMO) via the right axillary artery and right common femoral vein (Figure 1b). He was transferred to the intensive care unit (ICU), where high-dose inotropic and vasopressor agents were required. Right heart catheterization with EMB confirmed EM with myocyte necrosis (Figure 3). A broad infectious and autoimmune workup was initiated; relevant serologies were negative, and further genetic analyses were pending at the time.

High-dose intravenous methylprednisolone (1 g/day IV for three days) followed by oral taper and azathioprine (2 mg/kg/day orally) was commenced after multidisciplinary discussion with cardiology, oncology, and microbiology. Under immunosuppressive therapy, cardiac function gradually improved, with LVEF increasing to 40% on the ninth postoperative day, enabling uneventful explantation of ECMO on the same day.

**Figure 4:**
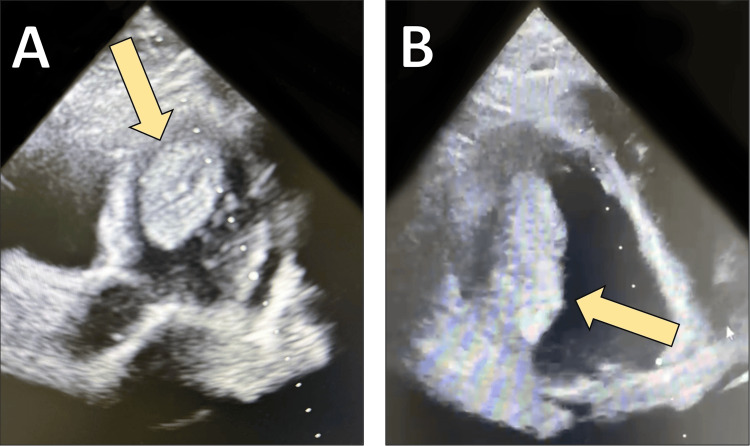
Transthoracic echocardiography demonstrating left ventricular thrombus A: four-chamber view revealing a large thrombus in the apical region of the left ventricle (yellow arrow); B: short-axis view confirming the presence and size of the apical thrombus (yellow arrow).

Despite recovery of cardiac function, the hospital course was complicated. Echocardiography revealed a large, highly mobile left ventricular thrombus; therefore, systemic anticoagulation with heparin followed by oral anticoagulation was initiated (Figure 4). The thrombus was no longer visualized on follow-up echocardiography.

On the 12th postoperative day, the patient developed signs of ileus. CT imaging showed diffuse colitis with possible ischemia. No occlusion of the mesenteric vessels was evident. Emergency laparotomy and right hemicolectomy with resection of the terminal ileum and part of the transverse colon were performed, resulting in a long Hartmann’s stump and end ileostomy. Histopathology confirmed ischemic colitis. Postoperatively, he required prolonged mechanical ventilation and was tracheostomized on the 17th postoperative day. Gradual weaning was successful, and decannulation was achieved by the 21st postoperative day.

During the subsequent ward stay, the patient developed a fever with positive blood cultures for Gram-positive cocci, treated with intravenous amoxicillin-clavulanate. Anticoagulation was transitioned from warfarin to apixaban due to concerns about compliance. He was discharged in stable condition on the 32nd postoperative day with instructions to continue methylprednisolone (gradual taper), azathioprine, and anticoagulation for at least six months (Figure 1c).

Six months later, the patient was readmitted for planned surgical restoration of bowel continuity. Dense adhesions were found intraoperatively, and adhesiolysis with ileostomy resection and side-to-side ileotransversostomy was performed without complications. The postoperative course was uneventful, with good mobilization, tolerance of oral intake, and normalization of inflammatory parameters. He was discharged one week after surgery on reduced doses of methylprednisolone and azathioprine, together with standard heart failure therapy, including a beta-blocker, an ACE inhibitor, and an SGLT2 inhibitor.

At follow-up, transthoracic echocardiography after seven months showed recovery of LVEF to 50%, with no evidence of recurrent thrombus. The patient remained in good general condition with no relevant symptoms.

## Discussion

Rarity and diagnostic challenges

EM is an uncommon inflammatory cardiomyopathy, but when it presents fulminantly, it carries a high risk of morbidity and mortality. Clinical manifestations are heterogeneous, ranging from mild dyspnea and chest pain to rapidly progressive cardiogenic shock and sudden cardiac death [1,2]. Peripheral eosinophilia, while sometimes present, is not a reliable diagnostic marker, and initial laboratory and imaging findings are often non-specific. CMR can raise suspicion by revealing subendocardial late gadolinium enhancement, but EMB remains the diagnostic gold standard, confirming both eosinophilic infiltration and myocyte necrosis [3]. In the presented case, EMB was critical in establishing the diagnosis and guiding treatment, reflecting the importance of histological confirmation emphasized in recent reviews [6].

Complications of eosinophilic myocarditis

The clinical course of EM is often complicated by severe and sometimes unexpected sequelae. Thrombus formation within the left ventricle is a well-described complication, attributed to endocardial injury, stasis from ventricular dysfunction, and eosinophil-mediated prothrombotic activity. In large cohorts, patients with EM experienced higher rates of systemic embolism compared to those with hypereosinophilic syndromes without cardiac involvement [7]. The patient described here developed a large and mobile apical thrombus that resolved only under systemic anticoagulation, underlining the need for early initiation of antithrombotic therapy in patients with impaired ventricular function.

Beyond cardiac complications, EM may also manifest with systemic ischemic events due to both low-output states and embolization. The ischemic colitis observed in this case exemplifies the potential for extra-cardiac organ injury, a complication rarely described but pathophysiologically plausible in the setting of cardiogenic shock and systemic inflammatory activation. Previous reports have highlighted gastrointestinal, renal, and neurological involvement in EM and in drug-related hypersensitivity syndromes such as drug rash with eosinophilia and systemic symptoms (DRESS) syndrome, suggesting that the disease can extend well beyond the myocardium [8]. These complications add complexity to the management of already unstable patients and demand multidisciplinary input.

To provide a clearer understanding of the clinical trajectory, the complications in this case can be divided into disease-related and treatment- or critical illness-related events. The left ventricular thrombus and cardiogenic shock were directly attributable to fulminant EM and its hemodynamic consequences. In contrast, complications such as infection, prolonged mechanical ventilation, and postoperative ileus/ischemic colitis were more likely related to the patient’s critical illness, prolonged ICU stay, and surgical intervention. Distinguishing between these categories helps contextualize the severity and systemic impact of fulminant EM.

Therapeutic approaches

Treatment of EM centers on rapid initiation of immunosuppressive therapy, with high-dose corticosteroids forming the cornerstone of management [3,4]. In patients with severe disease or steroid-refractory courses, additional agents such as azathioprine or cyclophosphamide are commonly added, and biologics targeting eosinophil pathways are being explored in refractory cases [9]. In the current patient, early combination therapy with methylprednisolone and azathioprine was associated with progressive improvement in left ventricular function, supporting the literature that underscores the benefit of aggressive immunosuppression when instituted promptly.

Equally important is supportive therapy. Patients presenting in cardiogenic shock often require advanced hemodynamic stabilization. ECMO has been successfully employed as a bridge to recovery in fulminant EM, allowing time for immunosuppressive agents to exert their effects. Several reports describe favorable outcomes in patients requiring prolonged ECMO support, with recovery of systolic function and survival to discharge [10]. In the present case, ECMO implantation was lifesaving, with cardiac function improving sufficiently to permit explantation after just over a week of support. This illustrates how MCS has transformed the prognosis of otherwise fatal cases.

Finally, anticoagulation plays a pivotal role in preventing and treating intracardiac thrombi, particularly in patients with impaired systolic function or visible thrombus formation [7]. While no standardized protocols exist, anticoagulation is widely recommended in such scenarios. Our patient required systemic heparinization and subsequent oral anticoagulation, with resolution of the thrombus confirmed on echocardiography.

Prognosis and future perspectives

Despite therapeutic advances, the prognosis of EM remains guarded. In a systematic review, hospital mortality reached nearly 20% overall and exceeded 30% in drug-associated hypersensitivity forms [1,9]. Survivors often recover left ventricular function, but the risk of recurrence, arrhythmias, and chronic myocardial damage persists, highlighting the need for close long-term surveillance. Cases of necrotizing EM, particularly those occurring after viral infections or vaccinations, have been associated with fulminant and often fatal courses, underlining the heterogeneity of this disease [11,12].

Future research is needed to clarify optimal immunosuppressive regimens, the role of biologic therapies, and the timing and selection criteria for ECMO initiation. Long-term follow-up data are sparse, and there remains uncertainty about the risk of late complications, including progression to dilated cardiomyopathy and sudden cardiac death. Nevertheless, this case illustrates that with timely diagnosis, prompt initiation of immunosuppressive therapy, MCS when indicated, and multidisciplinary care, survival with functional recovery is possible, even in fulminant and complicated presentations.

## Conclusions

EM is a rare but life-threatening condition that can progress rapidly to fulminant cardiogenic shock and multi-organ involvement. This case illustrates the importance of maintaining a high index of suspicion in young patients presenting with acute heart failure, as timely diagnosis by EMB and early initiation of immunosuppressive therapy can be lifesaving. The use of ECMO provided crucial circulatory support during the acute phase, while anticoagulation prevented embolic complications related to left ventricular thrombus. The occurrence of ischemic colitis in this patient underscores the potential for severe systemic complications and highlights the need for multidisciplinary management. Despite the guarded overall prognosis reported in the literature, this case demonstrates that with rapid recognition, aggressive therapy, and coordinated care, meaningful recovery of cardiac function and quality of life is achievable.
